# Acute Kidney Injury in a 31-year-old Male as a Consequence of Multiple Myeloma

**DOI:** 10.7759/cureus.2881

**Published:** 2018-06-26

**Authors:** Youngseo Lee, Kunal Goyal, Bhanu Prasad

**Affiliations:** 1 College of Medicine, University of Saskatchewan, Regina, CAN; 2 Radiology, Regina General Hospital, Regina, CAN; 3 Nephrology, Regina General Hospital, Regina, CAN

**Keywords:** acute kidney injury, myeloma, hypercalcemia, lytic lesions

## Abstract

We discuss a 31-year-old male who presented to the emergency room with a five-week history of dyspnea, chest pain, and right upper quadrant abdominal pain. Chest X-ray revealed a pleural opacity in the right lower hemothorax. Computed tomography (CT) of the chest showed a lytic rib lesion corresponding to the pleural lesion and multiple lytic lesions throughout the skeleton. Further labs revealed corrected calcium 4.43 mmol/L, total protein 115 g/L, creatinine 621 micromol/L, and urea 23.6 mmol/L. He had no prior labs for comparison. Subsequent bone marrow biopsy revealed a 50% involvement of plasma cells, which was consistent with a diagnosis of multiple myeloma (MM), and he was initiated on clone reduction therapy, with an excellent renal response but a partial hematologic response. This paper emphasizes that MM, though rare, should be in the differential diagnosis of acute kidney injury (AKI), as in this young adult.

## Introduction

Multiple myeloma (MM) is a condition characterized by the neoplastic proliferation of plasma cells in the bone marrow, leading to the excessive production of monoclonal immunoglobulin. Most common signs and symptoms include: Bone pain with lytic lesion on imaging modalities, elevated total serum protein concentration and/or presence of monoclonal protein in urine or serum, hypercalcemia, renal failure with urinalysis negative for blood and protein, and systemic signs and symptoms of malignancy (i.e. weight loss, night sweats, anemia) [1]. While MM is a disease of older adults, it should also be considered in the differential diagnosis in a younger individual with acute kidney injury (AKI).

## Case presentation

A 31-year-old male presented to the emergency department with a five-week history of right-sided chest pain, right upper quadrant abdominal pain, and associated shortness of breath. On initial clinical review, he claimed to be otherwise healthy with no prior medical or social history. The patient denied any prior history of renal disease. He claimed to have sustained a fall at work five weeks prior to presentation and started noticing gradually worsening right-sided chest pain. On initial presentation, he was tachycardic with a pulse rate of 104 beats/minute, blood pressure 121/76 mmHg, and oxygen saturation of 100% on room air. The cardiac examination was unremarkable with no additional sounds and murmurs. He was tender over lower four right ribs. Lungs were clear to auscultation with no rales or rhonchi. The abdomen was soft and non-tender, with no evidence of organomegaly, and there was no peripheral edema.

His important baseline investigations are listed in Table 1:

**Table 1:** Baseline investigations Ig: immunoglobulin

Test	Result	Range
Gamma globulin (g/L)	47.5	6.0-18.0
Total protein (g/L)	114	60-80
IgA (g/L)	0.79	0.57-3.94
IgG (g/L)	62.40	5.52-17.24
IgM (g/L)	<0.21	0.44-2.47
b2M (mg/L)	20.7	0.0-3.4
M spike (g/L)	44.6	
Kappa (k) (mg/L)	7.2	6.0-18.0
Lambda (l) (mg/L)	153.0	5.7-26.3
k/ l Ratio	<0.1	

**Figure 1:**
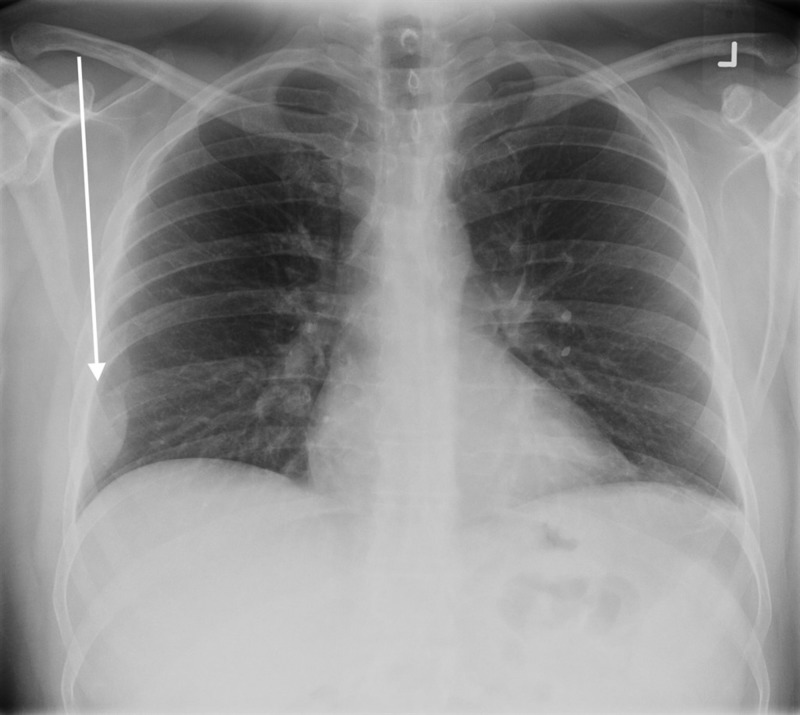
Pleural-based opacity identified within the right lower hemithorax. No rib fractures were identified. CT was recommended to evaluate the pleural lesion. CT: computed tomography

**Figure 2:**
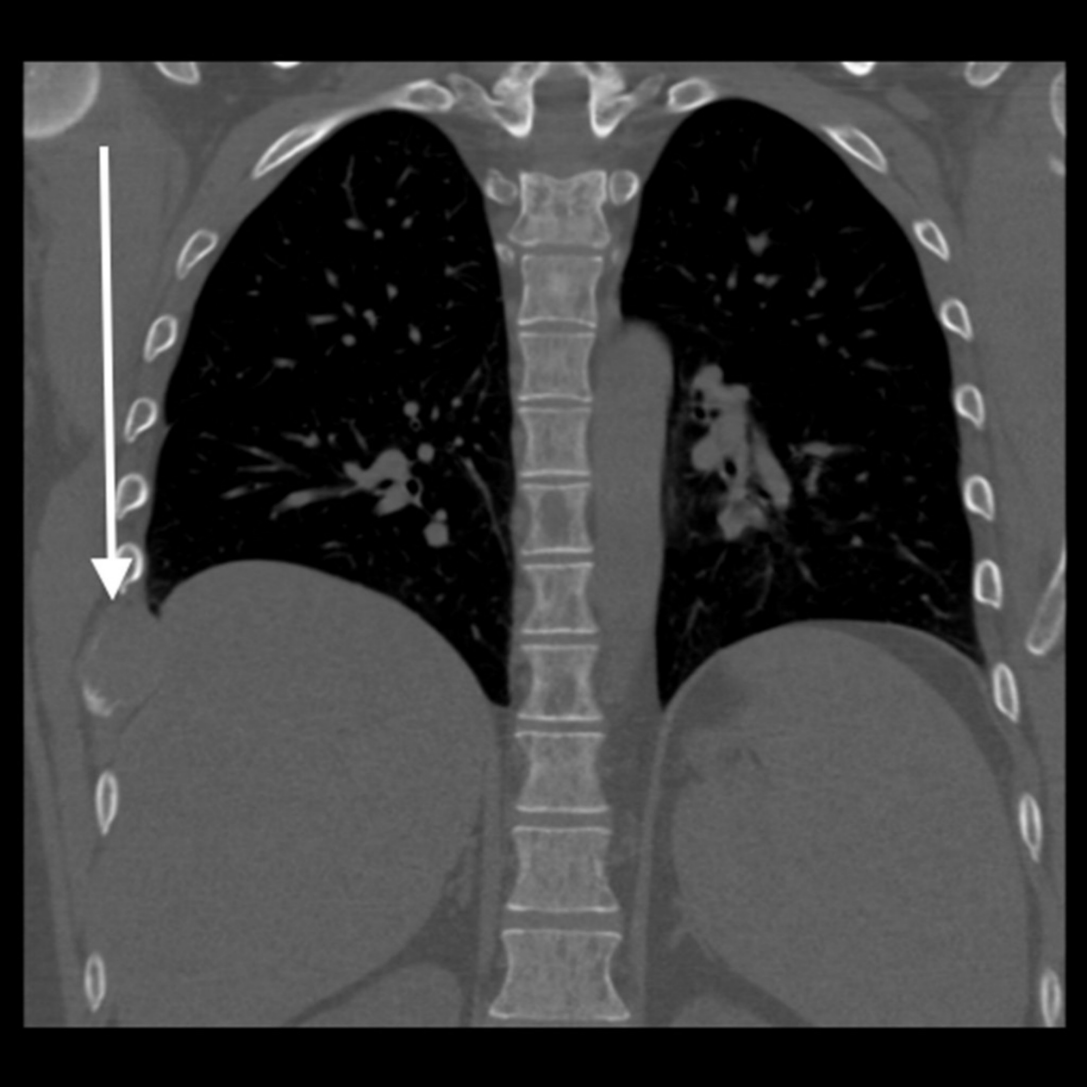
Coronal CT image on bone windows shows the lyric expansive rib lesion, corresponding to the chest x-ray abnormality. No fracture was seen. Pleura was normal. CT: computed tomography

**Figure 3:**
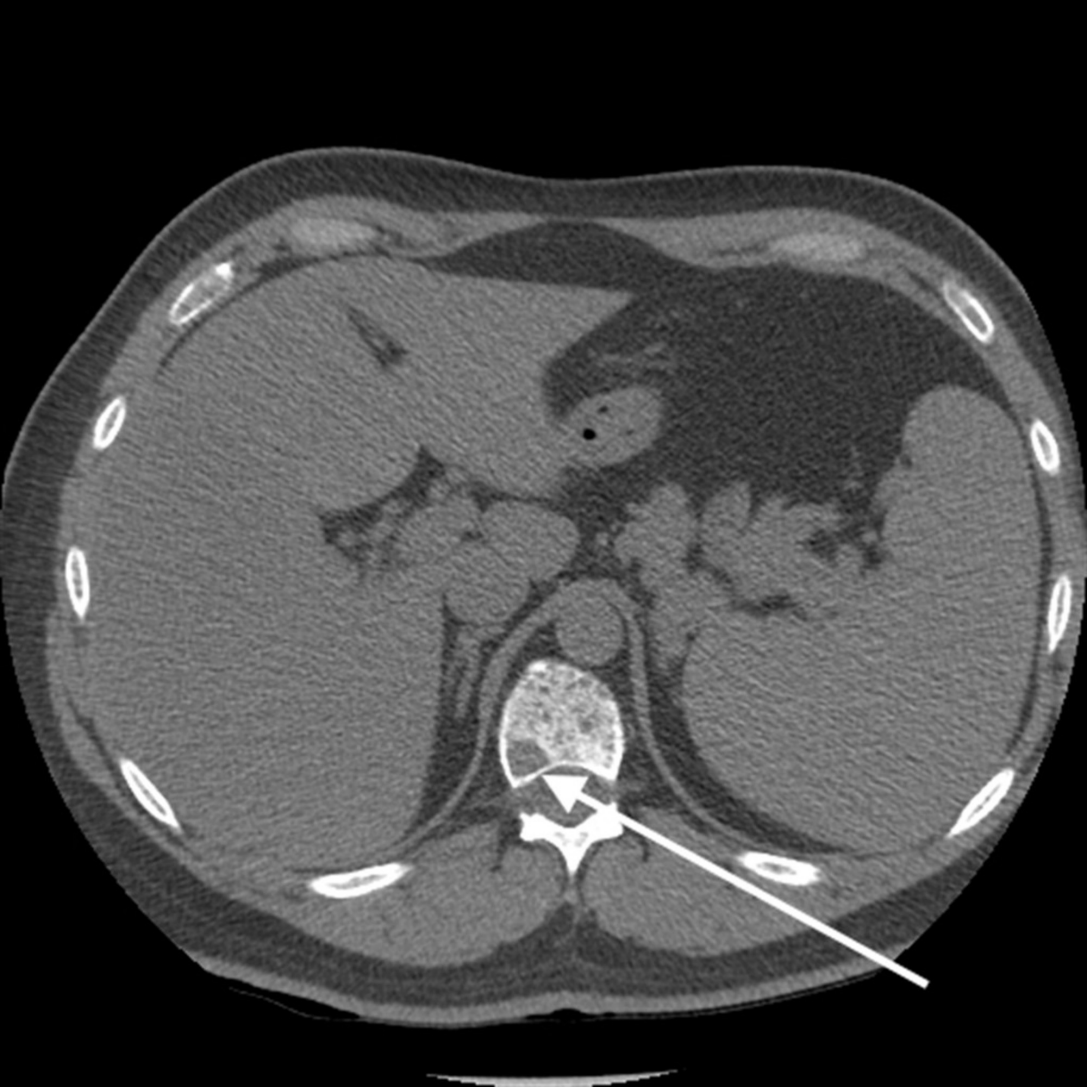
Lytic lesions were seen throughout the skeleton. Vertebral lesions are shown on axial and sagittal images. No pathological fracture seen within the spine.

**Figure 4:**
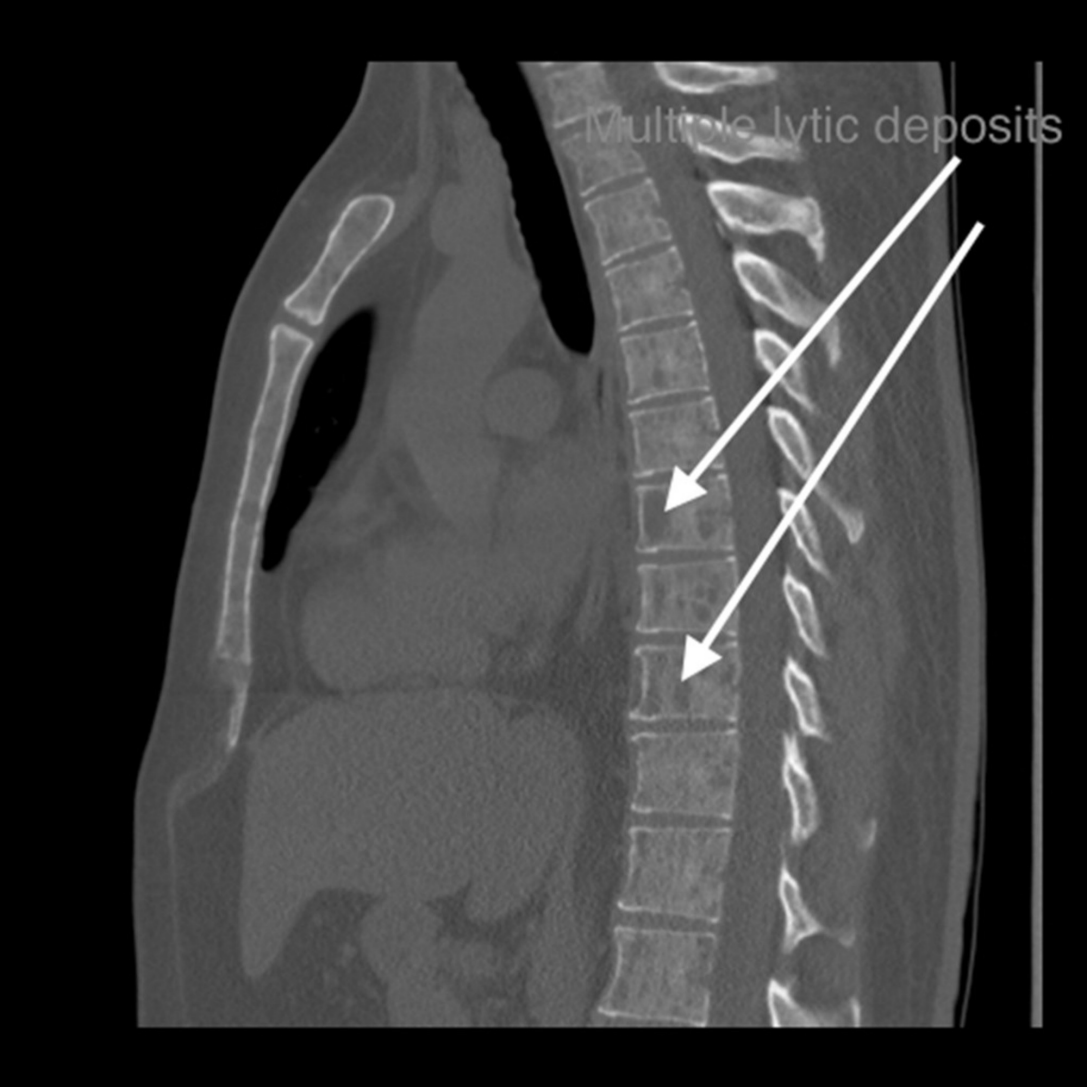
Lytic lesions were seen throughout the skeleton. Vertebral lesions are shown on axial and sagittal images. No pathological fracture seen within the spine.

He underwent a chest X-ray that suggested a pleural lesion in the right hemothorax (Figure 1). Computed tomography (CT) showed an expansile lytic lesion corresponding to the pleural lesion (Figure 2). Multiple lytic deposits were also seen on the CT scan (Figures 3-4). A subsequent skeletal survey revealed multiple lesions on the skull, pelvic bone, and proximal right femur. The lytic lesions were investigated further and revealed normocytic anemia with hemoglobin of 113 g/L, mean corpuscular volume (MCV) of 83.6 fL and 3+ rouleaux formation on peripheral blood smear, and normal white cell and platelet count. Corrected calcium was 4.43 mmol/L, and creatinine was 621 μmol with urea of 23.6 mmol/L. Urine microscopy was positive for protein and negative for blood.

On serum protein electrophoresis, total protein was 114 g/L with high gamma globulin 47.5 g/L (6.0-18.0 g/L) and a monoclonal band (M-spike) 44.6 g/L. Immunoglobulin panel revealed IgG 62.40 g/L (5.52-17.24 g/L), and immunofixation electrophoresis revealed monoclonal IgG, type kappa (k). Immunofixation of urine revealed Ig G (k) of 0.24 g/d. The patient consequently underwent a bone marrow core and aspirate biopsy of the iliac crest. The aspirate was suboptimal for evaluation for flow cytometry. The biopsy revealed sheets of plasma cells and interstitial infiltrate involving approximately 50% of the biopsy cellularity. Immunohistochemical staining was positive for CD 138 (suggestive of plasma cells). The results were consistent with a diagnosis of multiple myeloma based on diagnostic criteria by International Myeloma Working Group (IMWG).

He was started on the cyclophosphamide-bortezomib-dexamethasone (CyBorD) regimen and received four cycles of therapy. He had an excellent renal response but a partial hematologic response and relapsed after four months of therapy. The biochemical and hematologic response is documented in Table 2. He is now being treated with lenalidomide and dexamethasone, and once he achieves hematologic remission, he will undergo autologous stem cell transplant.

**Table 2:** Sequential changes in creatinine, corrected calcium, serum free light chains, and M-spike.

	Time of diagnosis CyBoRD	+1 month CyBoRD	+3 months CyBoRD	+4 months CyBoRD	+6 months lenalidomide initiated
Creatinine (mmol/L)	621	107	80	78	89
Calcium (mmol/L)	4.25	2.63	2.78	2.76	2.90
Free kappa (mg/L)	7.2	1.6	2.2	2.1	1.5
Free lambda (mg/L)	153	39.8	25.2	30.0	74.2
Kappa/lambda ratio	<0.1	<0.1	<0.1	<0.10	<0.10
M-spike (g/L)	47.5	26.1	19.7	17.6	28.7

## Discussion

MM is thought of as a disease of older adults. In a review of 1027 patients, who were newly diagnosed with multiple myeloma, by Kyle et al., the median age of diagnosis was 66 years, of which 2% were younger than 40 years and only 0.3% were under 30 years old [1]. There is limited information on young patients with MM due to a paucity of published data. There are a few isolated case reports on clinical presentation and outcomes. Consequently, consensus on population-specific clinical signs and symptoms, appropriate treatment, and prognostic factors is yet to be established.

Initial clinical features and laboratory investigations – including bone pain, fatigue, anemia, renal failure, and hypercalcemia – appear similar between patients under 40 years old and the general patient population of MM [1]. The proportion of patients with lytic bone lesions, frequency of associated systemic amyloidosis, and proportion of bone marrow plasma cells in immunofixation is also comparable with that in the general patient population with MM [2-3].

AKI, irrespective of age, in multiple myeloma is due to a multitude of causes.

The most common cause is light chain cast nephropathy. Acute or chronic kidney disease results from the overproduction and filtration of circulating toxic light chains, leading to tubular injury from intratubular cast formation and obstruction. The risk of light chain cast nephropathy is proportionally related to the urinary free light chain (FLC) concentration and the type of light chains. Light chain cast nephropathy generally occurs in the setting of high tumor burden and is uncommon in patients with low FLC concentrations (<500 mg/L). In our patient, the immunoglobulin G (IgG) (k) level in urine was only 0.24 g/day and is, therefore, unlikely to be the cause of AKI.

Another common cause of AKI is the concurrent use of intravenous contrast, diuretics, non-steroidal anti-inflammatory agents, and bisphosphonates. However, our patient wasn’t exposed to any of the above and, therefore, unlikely to have precipitated AKI.

Also, AKI has been reported to be due to acute tubulointerstitial nephritis from light chain deposition in the tubular basement membrane. This patient was not offered a kidney biopsy, but it is a rare cause and cannot be excluded.

Hyperviscosity is a rare but known cause of AKI. However, typically, it requires a higher M-spike (50-60 g/L) of IgG to cause hyperviscosity. In our patient, it was 47.5 g/L and there were no other clinical features of hyperviscosity and, therefore, it is unlikely to be a cause of AKI.

Hypercalcemia can contribute to the development of AKI by causing renal vasoconstriction, promoting intratubular calcium deposition through polyuria and volume depletion. In our patient, his calcium level was 4.4 mmol/L, his renal function started to improve with hydration and with an improvement in serum calcium, which leads us to wonder if his AKI was predominantly a consequence of hypercalcemia.

## Conclusions

In the absence of a kidney biopsy, the cause of AKI remains speculative, but the existing data does suggest that hypercalcemia was the main culprit in this young adult. Due to low frequency, a high index of suspicion is needed to diagnose younger patients with MM. However, it was reassuring to notice an excellent renal response to initial immunosuppressive therapy.
